# Efficacy and safety evaluation of neoadjuvant immunotherapy plus chemotherapy for resectable non–small cell lung cancer in real world

**DOI:** 10.3389/fonc.2022.1055610

**Published:** 2023-01-12

**Authors:** Min Fang, Qingqing Hang, Haitao Jiang, Lei Cai, Jinlin Hu, Hangjie Ying, Qing Gu, Xiaofu Yu, Jinshi Liu, Xiaojing Lai

**Affiliations:** ^1^ Department of Thoracic Radiotherapy, Zhejiang Cancer Hospital, Hangzhou, China; ^2^ Institute of Cancer and Basic Medicine (IBMC), Chinese Academy of Sciences, Hangzhou, China; ^3^ Zhejiang Key Laboratory of Radiation Oncology, Hangzhou, China; ^4^ The Second Clinical Medical College, Zhejiang Chinese Medical University, Hangzhou, China; ^5^ Department of Radiology, Zhejiang Cancer Hospital, Hangzhou, China; ^6^ Department of Thoracic Surgery, Zhejiang Cancer Hospital, Hangzhou, China; ^7^ Department of Pathology, Zhejiang Cancer Hospital, Hangzhou, China; ^8^ Zhejiang Cancer Institute, Zhejiang Cancer Hospital, Hangzhou, China

**Keywords:** NSCLC, neoadjuvant, immunotherapy, chemotherapy, surgery

## Abstract

**Objectives:**

The combination of immunotherapy and chemotherapy has shown great efficacy in stage IV non–small cell lung cancer (NSCLC) and is now widely used in clinical treatment strategy. This study retrospectively analyzed the efficacy and safety of neoadjuvant immunotherapy plus chemotherapy for resectable NSCLC in real world.

**Methods:**

We retrospectively analyzed patients with NSCLC who received neoadjuvant immunotherapy plus chemotherapy and underwent complete tumor resection in Zhejiang Cancer Hospital between January 2019 and January 2021. Tumor staging was based on the eighth TNM classification system of the American Joint Committee on Cancer staging criteria. The safety and toxicity (including operative and postoperative complications) and the efficacy [including objective response rate (ORR), disease control rate (DCR), tumor major pathological remission (MPR), and pathological complete response (pCR)] were evaluated.

**Results:**

In total, 368 patients with NSCLC were administered with neoadjuvant immunotherapy. Of them, 211 patients were included in this retrospective study. Most patients had stage II–III disease, with 75 (35.5%) and 88 (41.7%) patients diagnosed with clinical stages IIB and IIIA, respectively. A total of 206 patients (97.6%) received at least two doses of neoadjuvant immunotherapy plus chemotherapy. In addition, 121 patients (57.3%) have achieved MPR, and 80 patients (37.9%) have achieved pCR, with ORR at 69.2% and DCR at 97.7%. Treatment-related adverse events occurred in 46.4% of patients, and the incidence rate of grade 3 or 4 treatment-related adverse events was 13.3% (13/98). Moreover, adverse events of any grade of surgical complication occurred in 15.6% of patients. One-year disease-free survival was 80.6% (170/211).

**Conclusions:**

Neoadjuvant immunotherapy plus chemotherapy has significant efficacy with a high pCR and tolerable adverse effects for patients with resectable stage II–III NSCLC in real world.

## Introduction

Lung cancer is one of the leading causes of cancer-related deaths worldwide, and non–small cell lung cancer (NSCLC) accounts for 80%–85% of new cancer cases (1). Despite the combination of multimodal therapy treatment strategy including surgery, chemotherapy, and radiotherapy for patients with resectable NSCLC, 25%–70% of patients at different stages will relapse in 5 years (2). In the past decades, although many efforts have been made to develop the perioperative management of resectable NSCLC (3, 4), patients still have to face a high risk of recurrence and death. Therefore, it is still of urgent need to develop new treatment methods.

In past 5 years, immune checkpoint inhibitors (ICIs), especially programmed cell death 1 (PD-1) and programmed death ligand 1 (PD-L1) inhibitors, have significantly changed the treatment paradigm for patients with advanced NSCLC and provided long-term survival hope for patients with metastatic lung cancer. Now, PD-1 and PD-L1 inhibitors combined with chemotherapy have become the standard first-line treatment methods for advanced NSCLC (5–7). Given the profound impact of PD-1 and PD-L1 inhibitors on advanced NSCLC, many experts have paid great attention to investigating the potential role of ICIs in resectable NSCLC, and several undergoing clinical trials have reported promising results (8–11). The Checkmate 159 trial was the first study to use PD-1 inhibitor as neoadjuvant regimen for resectable NSCLC, and it showed that, after two doses of nivolumab preoperatively, 45% of resected tumors (9/20) had a major pathological remission (MPR), and 10% of patients (2/20) even achieved a pathological complete response (pCR) (12). The NADIM trial (NCT 03081689) applied three preoperative cylces of PD-1 inhibitor with chemotherapy on individuals with stage IIIA disease. The results showed that 41 patients had underwent tumor resection, 34 (83%) had achieved MPR, 26 (63%) had achieved pCR. Moreover, 37 patients (90%) achieved pathlogical downstaging, and 35 patients (85%) are alive and free of recurrence with a median follow-up of 24 months (9). Recently, the phase 3 Checkmate 816 trial showed that neoadjuvant with nivolumab and chemotherapy significantly improved the pCR (24.0%) compared with traditional chemotherapy (2.2%) for resectable NSCLC with a tolerable safety (13). All these data revealed that the neoadjuvant immunotherapy combined with chemotherapy may provide a new treatment strategy for resectable NSCLC.

In this study, we retrospectively collected data from 211 patients with resectable stage IB–IIIB NSCLC, who have received neoadjuvant immunotherapy plus chemotherapy and underwent complete tumor resection in our center to evaluate the efficacy and safety.

## Methods

### Patients and data collection

Patients with NSCLC who received neoadjuvant immunotherapy plus chemotherapy and underwent radical resection between January 2019 to January 2021 in Zhejiang Cancer Hospital were reviewed. A total of 211 patients with NSCLC identified from a screened population of 368 patients were enrolled in this study. The main inclusion criteria were as follows (1): histologically confirmed NSCLC (2), clinically stages I–III (3), no metastatic cervical lymph nodes or prior cancer therapy (4), negative driver mutation (5), received at least one dose of neoadjuvant immunotherapy plus chemotherapy, and (6) underwent radical surgery with curative intent. Tumor staging was based on the eighth TNM classification system of the American Joint Committee on Cancer staging criteria. All patients underwent routine baseline tumor diagnosis and staging, including chest computed tomography (CT), brain magnetic resonance imaging, and positron emission tomography–CT (PET-CT). The neoadjuvant regimen was PD-1 inhibitors combined with platinum-based chemotherapy, which was administered intravenously every 21 days. The PD-1 inhibitors include nivolumab, pembrolizumab, camrelizumab, toripalimab, sintilimab, and tislelizumab. Preoperative chest CT scan was necessary to evaluate the efficacy of neoadjuvant regimen. Follow-up information was obtained through inpatient medical records and telephone inquiries. The last follow-up date was 1 March 2022. This retrospective study was approved by the Institutional Ethics Board of Cancer Hospital of the University of Chinese Academy (No. IRB-2022-48).

### Study end points and assessment method

Radiological response of the tumor including objective response rate (ORR) and disease control rate (DCR) was assessed after neoadjuvant immunotherapy plus chemotherapy and before the operation according to the Response Evaluation Criteria in Solid Tumors version 1.1 (RECIST v1.1). Disease-free survival (DFS) was defined as the time from diagnosis to disease progression, relapse, or death, whichever came first.

Postoperative pathological remission including MPR and pCR was assessed by specilaized pathologist after neoadjuvant immunotherapy plus chemotherapy. MPR is defined as neoadjuvant therapy–induced tumor regression with less than 10% vital tumor tissue, and pCR is defined as neoadjuvant therapy–induced complete tumor regression without vital tumor tissue (14).

Neoadjuvant therapy adverse events were evaluated on the basis of the National Cancer Institute Common Terminology Criteria for Adverse Events version 5.0. From the beginning of neoadjuvant immunotherapy to the end of the treatment within 1 month, any adverse events that occurred, regardless of whether there is a relationship with the neoadjuvant immunotherapy, were judged as an adverse event. Time to surgery is defined as the time from the end of neoadjuvant therapy to the surgical operation. Postoperative complications occurred within 30 days after surgery were documented, including pain, anemia, subcutaneous emphysema, prolonged air leak, pneumonia, pleural effusion, and atrial fibrillation.

### Statistical analysis

Patients were characterized by clinicopathological variables such as age, sex, histology, and stage. Categorical variables were presented as absolute and relative frequency, and numerical variables were presented as mean (SD) or median. The median length of follow-up was calculated using the Kaplan–Meier method. The Kaplan–Meier method was also used to calculate the DFS. All the statistical tests were two-sided with a significance level at p<0.05. Statistical analyses were performed with the SPSS 25.0.

## Results

### Patients and treatments

From January 2019 to January 2021, 211 patients who were diagnosed with primary NSCLC underwent radical R0 resection after neoadjuvant immunotherapy plus chemotherapy in our center. The major clinicopathological characteristics of 211 patients were shown in **Table 1**. The patients were predominately male patients (196, 92.9%) and pathologically confirmed squamous cell carcinoma (172, 82%). Most patients were in stages IIB (75, 35.5%) and IIIA (88, 41.7%). Most of them (206, 97.6%) received at least two doses of immunotherapy plus chemotherapy. A total of 139 patients (65.9%) received adjuvant immunotherapy after surgery.

**Table 1:** Clinicopathological characteristics of 211 patients.

Characteristics	All patients (n, %)
Age, median (range), years	64 (38–77)
Sex Male Female	196 (92.9) 15 (7.1)
Smoking status Current/former Never	181 (85.8) 30 (14.2)
Histologic type of tumor Squamous Adenocarcinoma Other type/unknown	172 (81.5) 28 (13.3) 11 (5.2)
Disease stage at baseline	
IB	2 (0.9)
IIA	7 (3.3)
IIB	75 (35.5)
IIIA	88 (41.7)
IIIB	39 (18.5)
Doses of neoadjuvant immunotherapy 1 2 3 4	5 (2.4) 148 (70.1) 39 (18.5) 19 (9.0)
Adjuvant therapy^*^	
None	55 (6.1)
Chemotherapy	98 (46.4)
Immunotherapy	143 (67.8)
Radiotherapy	7(3.3)
T category downstaged	179 (84.8)
T category upstaged	13 (6.2)
N category downstaged	120 (56.9)
N category upstaged	17 (8.1)

^*^Eighty-nine patients received more than one adjuvant therapy.

### Surgery summary

**Figure 1:**
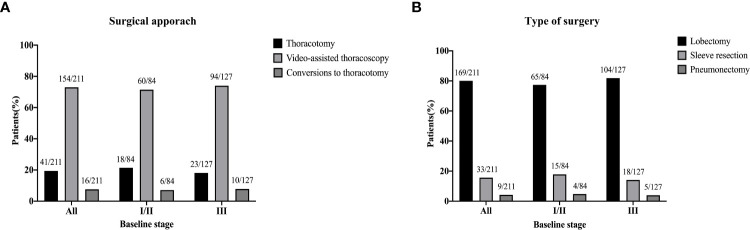
Surgical approach **(A)** and type of surgery **(B)** of patients by baseline stages of disease.

The median time to surgery was 4.1 (range, 0.9–17.4) weeks. The minimally invasive approach was more common, 154 patients (73.0%) underwent thoracoscopy surgery, 41 patients (19.4%) underwent thoracotomy, and 16 cases (7.6%) required conversion from thoracoscopy to thoracotomy. There are a total of 169 patients (80.1%) underwent lobectomy, 33 patients (15.6%) underwent sleeve lobectomy, and 9 patients (4.3%) underwent left pneumonectomy. The differences in surgical patterns of different cTNM stage were shown in **Figure 1**. The median length of hospitalization was 11 days (range, 5–31).

### Pathological assessment and efficacy

**Figure 2:**
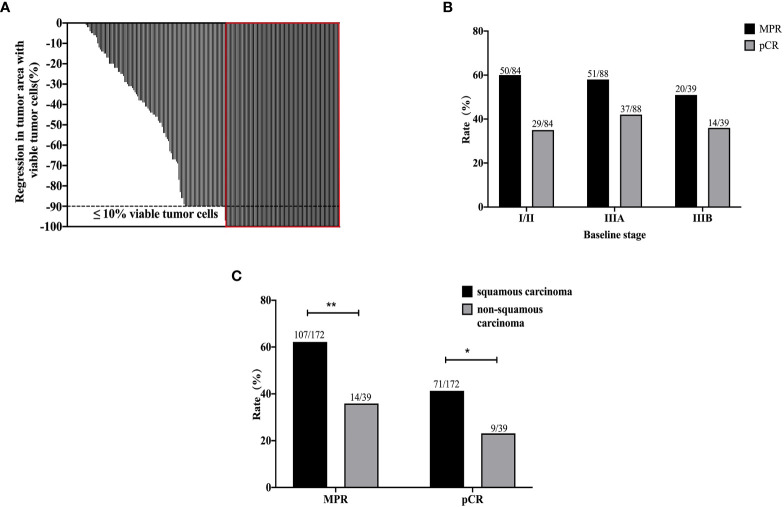
The pathological results of all 211 patients after neoadjuvant immunotherapy plus chemotherapy. The depth of pathological regression of all patients **(A)**. The MPR and pCR results by baseline stages of disease **(B)**. The MPR and pCR results of squamous carcinoma and non-squamous carcinoma **(C)**. **p < 0.01; *p < 0.05.

According to the RECIST v1.1, four patients achieved CR, 142 patients achieved PR (partial response), 60 patients achieved SD (stable disease), and 1 patient were evaluated PD (progression disease). In addition, four patients were unknown due to the lack of imaging data after neoadjuvant immunotherapy plus chemotherapy. The ORR was 69.2%, and DCR was 97.7%. A total of 179 patients and 120 patients experienced T downstaged and N downstaged, respectively (**Table 1**). According to the postoperative pathological results, the percentage of pCR and MPR was 37.9% (80/211) and 57.3% (121/211), respectively. The depth of pathological regression in the primary tumor was shown in **Figure 2A**. Among patients achieved MPR, 50 patients (41.3%) were in stage II, of which ypN0, ypN1, and ypN2 were 84.0% (42/50), 8.0% (4/50), and 8.0% (4/50), respectively; and 71 patients (54.1%) were in stage III, of which ypN0, ypN1, and ypN2 were 85.9% (61/71), 8.5% (6/71), and 5.6% (4/71), respectively. Among patients who achieved pCR, 29 patients (36.3%) were in stage II and 51 patients (63.7%) were in stage III (**Figure 2B**). More patients with squamous cell carcinoma could be observed in the MPR (χ^2^ = 8.998, p = 0.003) and pCR group (χ^2^ = 4.475, p = 0.034), with 71 patients (41.2%) who achieved pCR and 107 patients (62.2%) who achieved MPR (**Figure 2C**).

**Figure 3:**
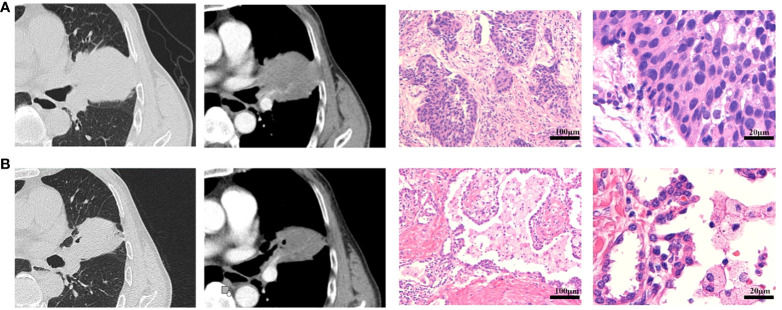
Radiological and pathological response of neoadjuvant immunotherapy plus chemotherapy. **(A)** The CT imaging and pathological diagnosis at baseline. **(B)** The CT imaging after two doses of neoadjuvant immunotherapy plus chemotherapy, and the pathological results after surgery. This was a 70-year-old male patient with smoking history, who was diagnosed as cT3N1M0 (stage IIIA) squamous cell carcinoma at baseline. After two doses of neoadjuvant immunotherapy plus chemotherapy, the patient achieved SD according to RECIST v1.1 with CT imaging assessment of 24% shrinkage of tumor. This patient underwent R0 resection with sleeve lobectomy and the pathological results with pCR. The regression bed is characterized by dense immune infiltrates with features of activation (tertiary lymphoid structure and dense tumor infiltrating lymphocytes infiltrates), along with features of cell death.

In addition, compared with the evaluation results of CT and postoperative pathology, the RECIST v1.1 evaluation based on preoperative CT imaging could not fully reflect the patient’s final pathological remission status. In addition, 1 patient who has been evaluated PD by radiologic assessment was confirmed to have no disease progression after surgery. Among 80 patients who achieved pCR, only four patients showed CR according to the RECIST v1.1, whereas 63 patients showed PR and 10 patients showed SD. The conformity between radiologic assessment and pathological assessment was 48.3% (102/211). The difference between the preoperative CT imaging and pathological evaluation results of a representative patient was shown in **Figure 3**.

At a median follow-up of 17.0 months, 1-year DFS was 80.6% (170/211). Twenty-eight patients have relapsed, and the specific progression patterns were shown in **Table 2**. In addition, 14 patients died during postoperative follow-up. Among them, six patients were related with tumor progression, three patients were dead within 30 days after surgery, three patients died with immune-related adverse events during the postoperative adjuvant immunotherapy, and another two patients died with unknown cause.

**Table 2:** The specific progression patterns of 28 patients.

Patient No.	cTNM	ypTNM	MPR or pCR	Progression pattern
3	cT_3_N_2_M_0_	ypT_1b_N_0_M_0_	–	Regional
7	cT_2b_N_1_M_0_	ypT_0_N_0_M_0_	pCR	Regional+Distant
9	cT_2_N_2_M_0_	ypT_0_N_2_M_0_	MPR	Regional+Distant
33	cT_2a_N_1_M_0_	ypT_0_N_2_M_0_	MPR	Regional
48	cT_3_N_2_M_0_	ypT_2b_N_0_M_0_	–	Regional+Distant
52	cT_3_N_0_M_0_	ypT_1a_N_1_M_0_	MPR	Regional
61	cT_3_N_2_M_0_	ypT_2a_N_1_M_0_	–	Regional
64	cT_3_N_0_M_0_	ypT_3_N_2_M_0_	–	Distant
79	cT_3_N_2_M_0_	ypT_3_N_1_M_0_	–	Regional+Distant
84	cT_1b_N_2_M_0_	ypT_0_N_0_M_0_	pCR	Distant
88	cT_3_N_2_M_0_	ypT_0_N_0_M_0_	pCR	Regional+Distant
101	cT_3_N_0_M_0_	ypT_2b_N_0_M_0_	–	Regional
112	cT_3_N_2_M_0_	ypT_1a_N_2_M_0_	MPR	Regional+Distant
114	cT_4_N_0_M_0_	ypT_4_N_0_M_0_	–	Regional
115	cT_3_N_2_M_0_	ypT_2a_N_0_M_0_	–	Regional+Distant
117	cT_3_N_0_M_0_	ypT_2a_N_0_M_0_	–	Distant
145	cT_3_N_2_M_0_	ypT_1a_N_0_M_0_	MPR	Regional+Distant
174	cT_2a_N_2_M_0_	ypT_4_N_2_M_0_	–	Regional
176	cT_2a_N_2_M_0_	ypT_2b_N_1_M_0_	–	Distant
177	cT_1c_N_2_M_0_	ypT_1b_N_2_M_0_	–	Regional
178	cT_4_N_2_M_0_	ypT_4_N_1_M_0_	–	Regional
186	cT_2b_N_2_M_0_	ypT_2_N_2_M_0_	MPR	Distant
188	cT_2a_N_1_M_0_	ypT_2b_N_2_M_0_	–	Regional
34*	cT_3_N_1_M_0_	ypT_2b_N_0_M_0_	–	Regional
41*	cT_4_N_1_M_0_	ypT_0_N_0_M_0_	pCR	Distant
62*	cT_4_N_2_M_0_	ypT_1c_N_0_M_0_	–	Distant
162*	cT_1b_N_1_M_0_	ypT_4_N_1_M_0_	–	Regional+Distant
208*	cT_1c_N_1_M_0_	ypT_2_N_2_M_0_	–	Distant

*indicates that the patients were dead.

### Safety and surgical complications

No previously unreported toxicities were observed in relation to the neoadjuvant immunotherapy plus chemotherapy. Overall, the incidence of treatment-related adverse events (TRAEs) was low, and most were grade 1 or 2. TRAE occurred in 46.4% of patients, and the incidence rate of grade 3 or 4 TRAE was 13.3% (13/98). The most common grade 3 or 4 TRAE was neutropenia (1.9%), immune-related hepatitis (1.4%), immune-related pneumonia (0.5%), thrombocytopenia (0.9%), and rash (0.9%) (**Table 3**). Among all these patients, 31 of them occurred more than two adverse events and six patients terminated the neoadjuvant immunotherapy due to the toxic effects.

**Table 3:** Treatment-related adverse events during neoadjuvant immunotherapy plus chemotherapy.

Adverse events, n (%)	Grade 1–2	Grade 3–4
Neutropenia	30 (14.2)	4 (1.9)
Decreased appetite	7 (3.3)	–
Fatigue	5 (2.4)	–
Nausea	4 (1.9)	–
Anemia	30 (14.2)	–
Rash	5 (2.4)	2 (0.9)
Increased aminotransferases	14 (6.6)	1 (0.5)
Thrombocytopenia	17(8.1)	2(0.9)
Pneumonia	2 (0.9)	1 (0.5)
Hepatitis	–	3 (1.4)
Fever	7 (3.3)	–
Arthralgia	5 (2.4)	–

Ninety-eight patients occurred treatment-related adverse events, of which 31 patients occurred more than two AEs (adverse events).

Adverse events in any grade of surgical complication occurred in 15.6% of patients. The most common adverse events were prolonged air leak (7, 21.2%) and pleural effusion (7, 21.2%) (**Table 4**). In addition, one patient experienced reoperation due to postoperative bleeding, and two patients experienced pulmonary embolism.

**Table 4:** Surgery-related adverse events.

Postoperative complications	n (%)
Pain	3 (1.4)
pneumothorax	7 (3.3)
Subcutaneous emphysema	2 (0.9)
Atrial fibrillation	1 (0.5)
Pleural effusion	6 (2.8)
Hypokalemia	4 (1.9)
Hyperkalemia	1 (0.5)
Postoperative bleeding	2 (0.9)
Anemia	2 (0.9)
Pulmonary embolism	2 (0.9)
Pulmonary atelectasis	1 (0.5)
Hoarseness	2 (0.9)
Pneumonia	7 (3.3)

Thirty-three patients occurred surgical complications, of which 10 patients occurred more than two AEs.

## Discussion

Neoadjuvant immunotherapy plus chemotherapy for resectable NSCLC is promising and attractive. This study is a retrospective real-world assessment of neoadjuvant PD-1 inhibitors plus platinum–based chemotherapy in patients with resectable stage I–III NSCLC. Neoadjuvant therapy given prior to radical surgery is usually conducted to downstage and improve the R0 resection rate in real world, and it had better compliance than adjuvant setting, with the biological effect that could be analyzed directly in the resected specimens (2). However, in the setting of neoadjuvant chemotherapy, the efficacy is relatively poor for NSCLC with pCR less than 4% (14). In addition, neoadjuvant chemotherapy just improved 5% of the 5-year survival rate on patients with resectable NSCLC with stage IB−IIIA (15). In our study, the combination treatment regimen with immunotherapy achieved significantly higher pathological response (MPR, 57.3%; pCR, 37.9%) compared with the historical neoadjuvant chemotherapy and tolerable adverse events. There are also several phase Ib/II clinical trials (9, 10, 12, 16–19), and a randomized phase III clinical trial (20) of neoadjuvant immunotherapy plus chemotherapy reported promising results. In the NADIM trial, the MPR rate was 83% (34/41). However, the initial results of the NEOMUN trial, which used pembrolizumab plus chemotherapy, reported that only four patients achieved MPR in 13 cases (17). Thus, the efficacy of neoadjuvant immunotherapy plus chemotherapy remained controversial based on the existing pilot studies, and more evidence is yet needed. The first reported phase III trial CheckMate 816 reported that neoadjuvant nivolumab plus chemotherapy significantly increased the MPR rates (36.9% vs. 8.9%, p < 0.05) and pCR rates (24.0% vs. 2.2%, p < 0.001) compared with neoadjuvant chemotherapy alone. Consistent with these clinical trials, higher percentage of pCR and MPR rate in squamous carcinoma group than that in non-squamous group was observed in our study with statistical significance (41.3% vs. 23.1% and 62.2% vs. 35.9%, respectively). In addition, patients with stage III NSCLC have the trend to benefit more from the combination treatment regimen than stage IB or II patients (pCR, 40.2% vs. 34.9%), which is consistent with previous reports of adjuvant chemotherapy (21).

National Comprehensive Cancer Network guidelines recommended four doses of adjuvant chemotherapy, whereas the dose of neoadjuvant immunotherapy plus chemotherapy is inconclusive. In general, most studies choose two to four doses, whereas CheckMate159 (12) and LCMC3 (19) trials chose two doses, NADIM (9) and CheckMate 816 trials were of three doses, and NCT02716038 trial (10) was of four doses. In addition, a meta-analysis showed that three doses of neoadjuvant chemotherapy could reduce the risk of death (15). In our study, more than two-thirds of patients received two doses of neoadjuvant of immunotherapy plus chemotherapy. In addition, 54.7% (81/148) of patients achieved MPR and 35.8% (53/148) achieved pCR. In the three or more doses subgroup, the percentage of MPR and pCR was 65.5% (38/58) and 44.8% (26/58), respectively. The results demonstrated that the increase of the neoadjuvant dose may have the trend to improve the pCR and MPR rate. In addition, preclinical studies suggested that there is a window between neoadjuvant immunotherapy and surgery, and shortening or delaying the interval between surgery and neoadjuvant immunotherapy could lead T cells to become inactivated or return to dysfunctional state, which will significantly affect survival (22). It is really challenging to determine the timing of surgery after neoadjuvant immunotherapy to ensure the strongest activity of T cells. In the NADIM trial, it is suggested to take operation 3 to 7 weeks after the end of neoadjuvant immunotherapy. In addition, the Checkmate 816 trial suggested to take operation within 6 weeks. An expert consensus for 2020 recommended to take operation 4 to 6 weeks after the last neoadjuvant immunotherapy (23). In this study, the median time between the end of neoadjuvant immunotherapy and surgery was 4.1 weeks. All patients underwent R0 resection, 73.7% of patients underwent minimally invasive surgery, and less than 10% patients received the conversion to thoracotomy. Moreover the addition of PD-1 inhibitors to neoadjuvant chemotherapy did not increase the incidence of surgery complications or impede the feasibility of surgery, as well as the length of hospitalization. These results indicated that the surgery timing in 4 to 6 weeks after the last neoadjuvant immunotherapy is practicable.

Notably, although studies have proposed MPR as a surrogate end point in neoadjuvant trials for resectable NSCLC (24–27), the relation between pCR and survival is still under debate in the setting of neoadjuvant immunotherapy. In the NADIM trial, the radiologic response according to CT scans and pCR was not significantly associated with survival (28). Unlike conventional chemotherapy, the response pattern of patients treated with immunotherapy may be different, with some patients developing pseudo-progression or hyperprogression (29). As in our study, the CT evaluation could not accurately reflect the efficacy of neoadjuvant immunotherapy, and recent studies showed that FDG PET-CT could better play the role in assessment of response to immunotherapy (30, 31). In addition, a recent study from the International Neoadjuvant Melanoma Consortium supports the role of pCR as an early surrogate end point for recurrence-free survival and overall survival (27). In our study, although we do not have the long-term survival data due to the short follow up, among the 28 patients who have progressed after surgery during follow up, only four patients were pCR, which may indicate that pCR may be related with better DFS. Thus, in this regard, it still need more trials and long follow-up to illustrate whether pCR is an appropriate surrogate end point. In addition, in the NADIM trial, PD-L1 expression could not predict survival (28), which was similar with the studies in metastatic NSCLC (5, 32). The SAKK 16/14 trial also demonstrated that there was no association between MPR and pretreatment PD-L1 expression (33). Thus, the PD-L1 expression was not mandatory in this study.

Overall, the preliminary results in this study showed the excellent efficacy of the neoadjuvant immunotherapy plus chemotherapy in resectable NSCLC. In addition, the addition of neoadjuvant of immunotherapy did not increase the difficulty of surgical procedure and surgery-related adverse events. However, there are some limitations in our study. It is a retrospective study from a single cancer center with short-term follow up, and there may be omissions in the records of immune-related and surgery-related adverse events. In addition, the PD-L1 expression status of patients at baseline and the patient reported outcomes were not recorded. This study only included patients who had undergone R0 resection after neoadjuvant immunotherapy, and the adjuvant therapy was not well controlled. Numerous questions still need to be investigated, such as the dose of neoadjuvant immunotherapy, the maintenance immunotherapy treatment after surgery, and the appropriate end point and biomarkers.

In conclusion, this study presented a promising efficacy of neoadjuvant PD-1 inhibitors plus chemotherapy for patients with resectable stage I–III NSCLC and tolerable toxicities. However, these findings still need prospective clinical trials to confirm.

## Data availability statement

The raw data supporting the conclusions of this article will be made available by the authors, without undue reservation.

## Ethics statement

The studies involving human participants were reviewed and approved by the Institutional Ethics Board of Cancer Hospital of the University of Chinese Academy (No. IRB-2022-48). Written informed consent for participation was not required for this study in accordance with the national legislation and the institutional requirements. Written informed consent was not obtained from the individual(s) for the publication of any potentially identifiable images or data included in this article.

## Author contributions

(I) Conception and design: MF, JL, and XL. (II) Administrative support: JL and XL. (III) Provision of study materials or patients: JL and XL. (IV) Collection and assembly of data: MF, QH, LC, HJ, HY, QG, and XY. (V) Data analysis and interpretation: MF, QH, HJ, and JH. (VI) Manuscript writing: MF and QH. (VII) Final approval of manuscript: All authors.
